# Draft Genome Sequence of *Bacillus mycoides* M2E15, a Strain Isolated from the Endosphere of Potato

**DOI:** 10.1128/genomeA.00031-16

**Published:** 2016-02-25

**Authors:** Yanglei Yi, Anne de Jong, Jan Spoelder, J. Theo M. Elzenga, Jan Dirk van Elsas, Oscar P. Kuipers

**Affiliations:** a Molecular Genetics, Groningen Biomolecular Sciences and Biotechnology Institute, University of Groningen, Groningen, the Netherlands; b Plant Physiology, Groningen Institute for Evolutionary Life Sciences, University of Groningen, Groningen, the Netherlands; c Microbial Ecology, Groningen Institute for Evolutionary Life Sciences, University of Groningen, Groningen, the Netherlands; d HLB Research & Consultancy in Agriculture, Wijster, the Netherlands

## Abstract

We present the draft genome sequence of *Bacillus mycoides* M2E15, a bacterium isolated from potato endosphere. Analysis of the 6.08-Mbp draft genome sequence identified 6,386 protein-encoding sequences, including potential plant growth promoting genes. Specifically, genes for proteins involved in phosphate utilization, iron acquisition, and bacteriocin production were identified.

## GENOME ANNOUNCEMENT

*Bacillus mycoides* is a rod-shaped chain-forming bacterium which is associated with the *Bacillus cereus* group. On agar plates, it forms unique rhizoid colonies resulting from cells that are linked end-to-end. Several reports on *B. mycoides* have described its plant-growth-promoting effects on sugar beet, cucurbits, and tobacco (1–3). We found *B. mycoides* to be present in the endosphere of potato without causing visible signs of disease. In order to understand the genetic make-up of this bacterium, we present the draft genome of the endophytic strain M2E15, which was isolated from the roots of potato (cultivar Miranda) in Wijster, the Netherlands.

Strain M2E15 was grown overnight in Luria-Bertani (LB) broth at 30°C, 200 rpm. Bacterial cells were harvested at the exponential growth phase and lysed with lysozyme. After RNase treatment, proteins were removed by proteinase K digestion. DNA was extracted from the lysate by phenol-chloroform treatment and recovered by isopropanol precipitation. Purified genomic DNA was sequenced using the MiSeq sequencing system of Illumina (4), yielding 250 bp paired-end reads with a mean library size of 400 bp. *De novo* assembly was performed using Velvet (4). Prediction of protein-encoding regions and automatic functional annotation was performed using the Rapid Annotations using Subsystem Technology (RAST) server (5). Moreover, the bacteriocin identification tool Bagel3 was used (6). The assembled genome of strain M2E15 consisted of 236 contigs with a total size of 6,077,838 bp. The chromosome harbors a total of 6,386 putative coding sequences (CDS) and 37 genes for tRNAs.

The *B. mycoides* M2E15 genome presents several genes that may be related to the solubilization of phosphate in soil. These include glucose dehydrogenase, lactate dehydrogenase, and citrate synthase, which are all involved in the production of organic acids. Organic acid production and release is considered to be a key mechanism for mineral phosphate solubilization. Moreover, a purple acid phosphatase gene, alkaline phosphatase and three 5′-nucleotidase genes were found, which may aid in the mineralization of organic phosphorus compounds (7). Several genes encoding proteins for the biosynthesis of exopolysaccharides were found in strain M2E15; these have considerable similarity with the *B. subtilis* eps operon, which is involved in biofilm formation, potentially aiding the organism in its interaction with plant roots (8). Genes for plant cell wall degradation enzymes, such as endoglucanase and glycoside hydrolase, are also present, suggesting its ability to degrade plant polymers (9, 10). Furthermore, the M2E15 genome was found to contain eleven chitinase genes, which may play a role in from the degradation of fungal and/or insect polymers (11). Finally, four bacteriocin gene clusters were predicted by BAGEL 3 (6).

### Nucleotide sequence accession numbers.

This whole-genome shotgun project has been deposited at GenBank under the accession number LLWA00000000. The version described in this paper is version LLWA01000000.
